# Regulation of Cytochrome *c* Oxidase by Natural Compounds Resveratrol, (–)-Epicatechin, and Betaine

**DOI:** 10.3390/cells10061346

**Published:** 2021-05-29

**Authors:** Icksoo Lee

**Affiliations:** College of Medicine, Dankook University, Cheonan-si 31116, Chungcheongnam-do, Korea; icksoolee@dankook.ac.kr; Tel.: +82-41-550-3972; Fax: +82-41-565-6167

**Keywords:** resveratrol, (–)-epicatechin, betaine, cytochrome *c* oxidase, oxidative phosphorylation, mitochondrial biogenesis

## Abstract

Numerous naturally occurring molecules have been studied for their beneficial health effects. Many compounds have received considerable attention for their potential medical uses. Among them, several substances have been found to improve mitochondrial function. This review focuses on resveratrol, (–)-epicatechin, and betaine and summarizes the published data pertaining to their effects on cytochrome *c* oxidase (COX) which is the terminal enzyme of the mitochondrial electron transport chain and is considered to play an important role in the regulation of mitochondrial respiration. In a variety of experimental model systems, these compounds have been shown to improve mitochondrial biogenesis in addition to increased COX amount and/or its enzymatic activity. Given that they are inexpensive, safe in a wide range of concentrations, and effectively improve mitochondrial and COX function, these compounds could be attractive enough for possible therapeutic or health improvement strategies.

## 1. Introduction

Mitochondria produce cellular energy, intermediates for biosynthesis, and reactive oxygen species (ROS) and play important roles in the regulation of metabolic homeostasis and cell death. Disruption of their function, dynamics, or biogenesis has been associated with a wide range of human diseases including cancer, diabetes, obesity, and aging [1].

The majority of cellular energy in the form of ATP is produced by mitochondrial oxidative phosphorylation (OXPHOS). This is carried out by the electron transport chain (ETC) complexes, which consists of respiratory chain complexes (complex I, II, III, and IV) and ATP synthase (complex V), which reside in the inner mitochondrial membrane. During electron transfer, complexes I, III, and IV (also known as cytochrome *c* oxidase; COX) pump protons across the inner mitochondrial membrane and create the mitochondrial membrane potential which is utilized by ATP synthase to produce ATP from ADP and P_i_. The OXPHOS complexes are also proposed to play a crucial role in maintaining cristae morphology, folded structures of the inner mitochondrial membrane [2]. In many species and tissues, the ETC complexes form supercomplexes with various stoichiometries, with respirasome (CI_1_CIII_2_CIV_1_) being the most well-known [2]. The significance of these supramolecular structures has been suggested to be functionally relevant to ETC activity by increasing the efficiency of NAD-linked respiration and by reducing ROS production [3]. Furthermore, disruptions in supercomplex organization have been associated with human diseases including congenital mitochondrial disorders, senescence, and other age-related diseases [4].

The efficiency of OXPHOS is controlled by mitochondrial dynamics as well. Although there are tissue and cell type-specific differences, mitochondrial size and shape continuously change by fusion and fission processes dependent on cellular bioenergetic requirements. These dynamic events also facilitate the maintenance of mitochondrial homeostasis and quality control [5]. Fused mitochondrial networks found in energetically demanding conditions allow mitochondria to mix their components, including mitochondrial DNA and OXPHOS complexes, to maintain efficient bioenergetic capacity [6,7]. In contrast, the fission process induces fragmentation of mitochondria that are often present in resting cells with low energetic demand and participate in the removal of dysfunctional ones.

Cytochrome *c* oxidase (COX, complex IV), the last step of the ETC receives electrons from cytochrome *c* and irreversibly reduces oxygen to water. Mammalian COX is dimeric and contains 13 tightly bound subunits as confirmed by X-ray crystallography [8]. Subunits, I, II, and III are encoded by the mitochondrial genome and among them the biggest two, subunit I and II fulfill the catalytic reaction of COX. The remaining 10 subunits, IV, Va, Vb, VIa, VIb, VIc, VIIa, VIIb, VIIc, and VIII are encoded by the nuclear genome and regulate the enzyme [9]. Therefore, the biogenesis of COX is indeed a finely concerted and synchronized process of both genomes [10]. Recently, NDUFA4, which had originally been considered as a subunit of complex I, was suggested to be a loosely bound subunit of COX [11]. Previously, this subunit had not been detected in a holoenzyme by X-ray crystallography and SDS-PAGE, since it is lost during the traditional COX purification process.

Considering the significance of the crucial role that COX plays in aerobic energy metabolism by controlling mitochondrial respiration, COX must be tightly regulated. Indeed, several regulatory mechanisms are known, such as an allosteric regulation, expression of tissue-/species-/development-specific isoforms, reversible phosphorylation modification via cell signaling, protein-protein interactions, and supercomplex formation [9,12]. These regulatory mechanisms affect the enzymatic activity of COX, which in turn alters mitochondrial membrane potential, and therefore ATP and ROS production. It has been reported that COX dysfunction is associated with many diseases where energy and ROS production are found to be dysregulated [13].

Many natural compounds that plants synthesize to reduce damage and increase their survival in response to environmental stresses, such as UV light, malnutrition, dehydration, pathogenic infection, and predators, are known to have anti-oxidative and anti-inflammatory properties [14]. Their biological activities are found to be beneficial for human health by diminishing or preventing pathological conditions that are associated with oxidative stress. Interestingly, a good number of these compounds affect mitochondrial function, including OXPHOS, by regulating mitochondrial biogenesis, via allosteric control, and possibly cell signaling [15,16,17,18]. Among them several compounds, such as resveratrol (RSV), (–)-epicatechin (EPI), betaine (BET), curcumin [19,20,21,22,23,24], and quercetin [25,26,27,28,29], have been shown to have an effect on COX. It has been reported that they increase protein amount and enzymatic activity of COX in various cell types and more pronounced effects were obtained under challenged or stressed conditions. Targeting and modulating COX by these compounds may therefore serve as a potential therapeutic approach since it could counteract COX dysfunction, which is observed in many pathological conditions. In this report, the most in-depth studied compounds, RSV, EPI, and BET, that affect COX are reviewed.

## 2. Resveratrol

**Figure 1:**
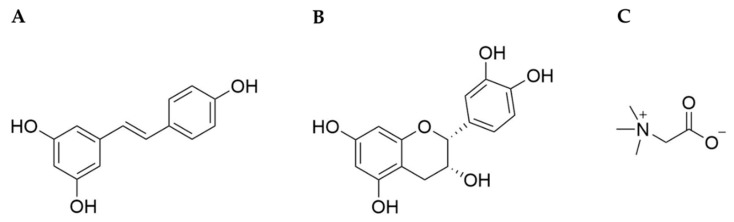
Chemical structures of resveratrol (**A**), (–)-epicatechin (**B**), and betaine (**C**).

Resveratrol (3,4’,5-trihydroxystilbene, RSV, Figure 1A) is a polyphenolic phytoalexin and synthesized in plants as a defensive mechanism responsive to UV light, microbial infection, pesticides, and others [30]. Its synthesis starts from a phenylpropanoid pathway, in which phenylalanine or tyrosine are converted into *p*-coumaroyl CoA, which is in turn condensed with malonyl CoA by stilbene synthase, producing RSV [31]. It was first isolated from *Veratrum album*, a type of grandiflorum, and characterized in 1939 [32]. It is found in various edible plants, such as peanuts, grape skins, berries, cacao, and others [33]. When consumed orally, it is converted into resveratrol sulfate and resveratrol glucuronide conjugates [34,35].

Initially, RSV drew attention for its possible role in the “French paradox” phenomenon, the epidemiological observation that French people have a lower incidence of coronary heart disease despite a diet rich in saturated fats [36], and its beneficial effects have been demonstrated to include anti-oxidative, anti-inflammatory, anti-apoptotic, and anti-cancer properties [37,38,39,40,41]. Due to the nature of its structure, RSV efficiently scavenges ROS and suppresses lipid peroxidation, and in addition, it exerts the anti-oxidative action indirectly by increasing expression of ROS scavenging enzymes [15]. There have been a number of studies showing that RSV enhances muscle performance [42,43,44,45] and alleviates or even prevents cardiovascular disease, neurodegenerative disease, cancer, and metabolic defects [46,47,48,49]. The compound is generally considered non-toxic, excluding mild adverse effects such as nausea, stomach pain, bloating, and diarrhea at high dosages (>1000 mg/day) [50]. RSV has been applied widely as a dietary supplement and active ingredient in cosmetic and dermatological products [51].

One of the molecular targets of RSV responsible for its protective properties is sirtuin 1 (SIRT1), a class III NAD^+^-dependent histone deacetylase in the nucleus. SIRT1 regulates several transcription factors and co-regulators for numerous physiological processes including glucose and fat metabolism, insulin production, and cell survival [52,53]. The intimate relationship between SIRT1 and mitochondria is possible through the action of a transcription coactivator, peroxisome proliferator activated receptor gamma coactivator 1 alpha (PGC1α) [54]. PGC1α is known to play a crucial role in mitochondrial energy metabolism and biogenesis by regulating transcription factors, such as NRF1/2, ERRα/β/γ, PPARα/γ/δ, FOXO1, and others [55,56]. It has been shown that the effects of RSV administration [57,58] are similar to those of caloric restriction in that both regimens share the same upstream regulating mechanism of SIRT1 activation and induction of PGC1α [59]. Another important regulator of PGC1α is AMP activated protein kinase (AMPK), a key metabolic energy sensor and regulator [60]. Interestingly, AMPK and SIRT1 are mutual regulators of each other, and they both play pivotal roles in regulating glucose homeostasis and mitochondrial biogenesis in response to stress and nutrient status [61,62,63]. The mediators of mitochondrial biogenesis induced by RSV are reviewed in detail in [15].

Enhanced mitochondrial biogenesis by RSV through SIRT1/PGC1α was consistently and reproducibly observed in liver, skeletal muscle, brown adipose tissue, brain, and other tissues [53,57,64,65,66]. Lagouge et al. showed that RSV (400 mg/kg per day for 15 weeks) induced increased gene expression of components that are involved in mitochondrial biogenesis [57]. The increase in mRNA levels of COX subunits (COX IV-1, COX Va, COX Vb, COX VIIaL) were confirmed along with those of other OXPHOS complex genes, such as NDUFB8 and ATP5G3 (Table 1). This enhancement could account for the increased muscle function seen in running endurance tests of high fat diet-fed mice with RSV administration.

A moderate dose of RSV in high fat diet-fed mouse skeletal muscle and hepatocytes (25–30 mg/kg per day for 8 months) and in C2C12 cells (25 μM for 24 h) increased AMPK activation, mitochondrial biogenesis, and mitochondrial function in a SIRT1 dependent manner [62]. In this study, mRNA levels of PGC1α, NRF, and subunits of OXPHOS complexes (NDUFS8, SDHB, UQCRC1, COX Vb, ATP5A1), ATP levels, mitochondrial mass, mitochondrial respiration, and membrane potential were all improved by RSV administration. The enhanced mitochondrial biogenesis by RSV was observed in nerve cells as well. A study of neurobehaviorally deficient rats found that intragastric administration of RSV (30 mg/kg per day) ameliorated disrupted mitochondrial features, such as reduced mitochondrial biogenesis, membrane potential, and ATP content, and elevated ROS level in hippocampal cells [71]. In a study of rats with status epilepticus, 100 μmol RSV microinjection into the hippocampus augmented mitochondrial content and protected the cells from apoptosis [67]. The increase in mitochondria after RSV injection was mediated by PGC1α-NRF1-mitochondrial transcription factor TFAM, and the levels of COX subunit I and mitochondrial DNA increased as a result of enhanced mitochondrial biogenesis. Another study with aged rats showed the neuroprotective effect of RSV alone and in combination with soy isoflavones [68]. RSV (80 mg/kg per day for 12 weeks) relieved the aberrant features of hippocampal mitochondria, such as swelling and vacuolization, and decreased apoptosis. Both protein levels of COX subunit I and antioxidant enzyme activity were restored following RSV administration.

The upregulation of COX might be explained through the regulation of COX assembly factors. Indeed, the expression of COX18, which is required for the translocation of the C-terminus of COX subunit II across the mitochondrial inner membrane [72], was reported to be increased by RSV [73]. RSV administration (500 mg per day) together with a 12-week exercise regimen produced a synergistic improvement of muscle capacity (i.e., improved oxygen uptake and muscle fatigue resistance) and mitochondrial density in 65–80-year-old men and women. In this group, gene expression levels of COX18 were increased three-fold, along with other mitochondrial metabolic genes following RSV treatment combined with exercise.

While the majority of literature demonstrates RSV’s effect on OXPHOS via mitochondrial biogenesis, another regulatory mechanism of RSV has been proposed. It has been suggested that RSV binds directly to complex I [74], III [75], and V [76,77,78] and alters their activities, yet no study has thus far shown that COX is a target of RSV. One to five μM RSV stimulated the activity of isolated complex I when co-incubated, which in turn increased the NAD^+^/NADH ratio [74]. In this study, enzymatic activities of complex I, II, and III of HepG2 cells were enhanced by RSV even though overall amounts of mitochondrial proteins did not change. In rat brain mitochondria, high concentrations of RSV (100 μM) competed with coenzyme Q for the substrate binding site on complex III and consequently suppressed the enzymatic activity by ~20% [75]. Another target affected by RSV is F_0_F_1_-ATPase (complex V). Incubation of pico to nano molar concentrations of RSV slightly activated F_0_F_1_-ATPase activity by ~10% in rat liver mitochondria but not in heart mitochondria [77], while RSV at higher concentrations (>1 μM) inhibited its enzymatic activity in rat brain and liver [77,78].

Free radical scavenging and anti-oxidative activities are well-known properties of RSV [79] and suggested to protect mitochondrial ETC complexes from oxidative stress. Incubation of rat brain homogenates with metabolites citrulline or ammonia, which were employed to mimic a urea cycle disorder, citrullinemia type 1, induced the deterioration of mitochondrial energy metabolism by downregulating complex II and COX [69]. The suppressed activities of these two enzymes were recovered by co-incubation with sub-millimolar concentrations of RSV for 1 h. Regarding the underlying mechanism, the authors proposed that anti-oxidative action of RSV protected the ETC complexes from ROS produced by the citrulline or ammonia treatment.

Conversely, some literature suggests that RSV may induce ROS production [80]. RSV has been proposed to inhibit cell proliferation, growth, angiogenesis, and cancer metastasis of several cancers, such as breast, lung, glioma, and prostate [81,82,83,84] and to potentiate the efficacy of chemotherapeutic agents [85,86,87,88]. The cell damage and eventual cell death by ROS-induced damage via modulating ETC complexes might be an anti-cancer mechanism of RSV. RSV-induced cell death mediated by elevated superoxide production was observed in CEM, a lymphoblastic leukemia cell line [70]. The cytotoxic effect of RSV in the leukemia cells was blunted by overexpression of Bcl-2 (CEM/Bcl-2), accompanied by increased COX activity and mitochondrial respiration. The elevated COX activity and mitochondrial respiration in the CEM/Bcl-2 declined after RSV incubation for 6 h in a dose-dependent manner up to 50 μM and was not due to the changes in COX amounts. However, no effect of RSV on COX was observed in control CEM cells. The apoptotic effect of RSV on SW620 colon cancer cells seemed to be triggered by increased ROS production due to elevated mitochondrial respiration [41]. Along with elevated oxygen consumption and mitochondrial metabolic activity, COX activity was increased by ~45% and ATP content was increased by ~35% after 10 μM RSV treatment for 48 h compared to control. This enhanced mitochondrial function might be due to the increased mitochondrial content, and mitochondrial mass was indeed increased. Moreover, protein levels of all OXPHOS complexes except complex III were increased, and among them, COX was the most affected by RSV treatment. However, unlike the studies discussed above, not SIRT1, but SIRT3, which resides inside the mitochondria, was associated with the RSV effect, accompanied by elevation of transcription factors for mitochondrial biogenesis, such as PGC1α, NRF1, TFAM [41]. Intriguingly, a recent study suggested that COX is a novel target of SIRT3, which led to COX activation in rat brain and K13, K264, K319, and K481 on COX subunit I were identified as deacetylation sites for SIRT3 [89].

The initial idea of using RSV to extend lifespan originated from studies with *Saccharomyces cerevisiae*, mediated by stimulating silent information regulator (Sir) [90]. Since then, RSV has been extensively investigated for the use in higher organisms including humans. RSV successfully prolonged life spans of unicellular organisms and some animals such as *Caenorhabditis elegans* [91,92,93] and *Drosophila melanogaster* [91,94]. However, its efficacy in mammals turned out to be virtually insignificant [95,96,97]. Rather than being a longevity wonder drug, RSV has been explored for its beneficial effects on human disease models [95]. Based on its functions regulating mitochondria and ROS, RSV has been proposed to be a possible treatment regimen for OXPHOS deficiency alone or in combination with other therapeutic substances. Papepe and Coster listed the studies evaluating RSV treatment in cultured skin fibroblast from patients with OXPHOS defects that are associated with genetic mutations [98]. The remedial values of RSV on COX deficiency caused by the mutation of COX assembly factors such as SURF1 and COX10 varied from positive to no effect [99,100,101]. It seems that RSV may be useful to treat certain defects and conditions but not others.

## 3. (–)-Epicatechin

(–)-epicatechin ((2*R*,3*R*)-2-(3,4-dihydroxyphenyl)-3,4-dihydro-2*H*-1-benzopyran-3,5,7-triol, EPI), a monomeric flavan-3-ol is a non-glycosylated flavonoid, and is found in rich quantities in cacao, tea, peanuts, apples, grapes, and berries [102]. It is suggested that EPI provides plants protection from microbial pathogens and predators by causing toxicity and interference in digestive enzymes. EPI is synthesized by phenylpropanoid and flavonoid biosynthetic pathways. It is produced by anthocyanin synthase and anthocyanin reductase from leucoanthocyanidin, which is derived from flavanone through a series of enzyme-catalyzed reactions [31]. EPI is one of four diastereoisomers of catechin that contains one dihydropyran and two benzene rings with five hydroxyl groups (Figure 1B). Due to its phenolic hydroxyl groups, EPI can efficiently scavenge free radicals [103]. It has the highest bioavailability in plasma and urine among other catechins [104], and is metabolized by conjugation with glucuronides, sulfates, and O-methyl sulfates once ingested [105].

EPI has been reported to guard cells from oxidative stress by several means [106]. Anti-oxidative efficacy of EPI is mediated by direct interaction with and removal of ROS and reactive metal ions. As an indirect anti-oxidant, EPI upregulates anti-oxidant enzymes, downregulates pro-oxidant enzymes, and produces phase II detoxifying/anti-oxidant enzymes. In addition, it suppresses inflammation by modulating oxidative stress-related cell signaling pathways [106]. Because of its anti-oxidative properties, EPI has been proposed to prevent or even rescue from diseases which are caused by or associated with oxidative stress, such as cardiovascular disease, neurodegenerative disease, diabetes, obesity, cancer, and others [107,108,109,110,111].

The fact that Kuna Indians in the San Blas Islands of Panama, who consume lots of cocoa drinks, have low incidence rates of hypertension and cardiovascular disease [112] evoked interest in the protective effect of EPI, since it is the most abundant polyphenolic monomer in cacao [113]. The administration of EPI rich cocoa has been suggested to improve the gastrointestinal system, nervous system, muscle performance, plasma lipid profiles, glucose homeostasis, obesity, and insulin sensitivity [114,115,116,117,118,119,120,121,122,123,124]. Many of these advantageous effects have been validated by the administration of pure EPI compound in humans and rodents [125,126,127,128,129,130,131,132,133,134,135,136,137,138].

Mounting evidence suggests that these beneficial effects of EPI might be facilitated by enhanced mitochondrial mass, integrity, and function (extensively reviewed in [125]). Several studies have reported the enhanced mitochondrial biogenesis in response to EPI was mediated by SIRT1-PGC1α [138,139,140] and nitric oxide [138,140,141], and considerable changes in protein levels and function of OXPHOS complexes have been reported in animals and cultured cells [125]. Direct modulation of mitochondria by EPI has been suggested to increase their function and integrity. EPI treatment of isolated rat heart mitochondria stimulated mitochondrial state 2 respiration in a dose-dependent manner and suppressed cytochrome *c* release, indicating stabilized integrity of mitochondrial membranes [142]. Its positive effect on membranes was further supported by an increase in cristae formation in skeletal muscle of patients with type 2 diabetes and heart failure [140] and with Becker muscular dystrophy [143], and in mouse heart and muscle [132].

EPI effects on OXPHOS complexes, particularly with regard to COX, have been investigated most extensively in muscle cells [144,145,146,147,148], along with endothelial cells [129], β cells [149], fat cells [150], and some cancer cells [151] (Table 2).

Moreno-Ulloa et al. showed increased mitochondrial biogenesis by 10 μM EPI treatment for 48 h with increased TFAM, NRF2, and porin levels, and citrate synthase activity in differentiated C2C12 cells [144]. In accordance with the increased mitochondrial mass, COX subunit I levels increased as well, while no significant change in complex II was reported. A possible contribution of a G-protein coupled estrogen receptor as an EPI receptor on the plasma membrane in skeletal muscle was proposed for mitochondrial biogenesis since the effect of EPI was blocked by inhibition of the receptor. Several rodent studies revealed that EPI has a positive effect on exercise performance, mitochondrial biogenesis, and function in skeletal muscles similar to the results of an exercise regimen. EPI administration (1.0 mg/kg, twice a day) stimulated mitochondrial biogenesis in mouse skeletal muscle, and its combination with 8 weeks of running exercise brought out cumulative results [148]. The enhanced exercise performance by EPI might be explained by the increased angiogenesis and mitochondrial biogenesis within the muscle. Increased capillary development was accompanied by the upregulation of pro-angiogenic factor VEGFR2 and downregulation of anti-angiogenic factors, such as ADAMTS1 and TSP1, along with suppressed FOXO1 levels. Expression levels of mitochondrial biogenesis regulators PGC1β and TFAM were upregulated and concurrently citrate synthase activity, which is often used as an indicator for mitochondrial content, was increased by EPI administration. Not only does EPI reinforce exercise performance by increased mitochondrial biogenesis and angiogenesis, it can also attenuate the loss of exercise benefits due to detraining [145]. Enhanced features in mouse hind muscles from 5 weeks of endurance training included increased running distance and time, capillarity, amount of complex V and COX, and COX activity. These adaptations reverted to the pre-exercise state 14 days post exercise cessation; however, EPI intake (1.0 mg/kg, twice a day) attenuated the regression and retained the adaptations to an almost similar extent as the exercised state. Among OXPHOS complexes, COX was the one most influenced by EPI with increases in the amount and activity of COX by training (~35% and ~144%, respectively) and those maintained by EPI administration during detraining (~45% and ~108%, respectively) compared to control. A similar beneficial effect of EPI on skeletal muscles against exercise cessation occurred in muscle atrophy induced by hindlimb suspension [146]. EPI administration (1 mg/kg, twice a day for 14 days) diminished the muscle degeneration caused by hindlimb suspension and improved the angiogenesis and mitochondrial biogenesis along with COX amount.

In contrast to the above studies, a clinical trial assessing the effect of EPI on the aerobic training adaptation in heathy human subjects turned out to be disappointing [133]. EPI supplementation (100 mg, twice a day) during 4 weeks of cycle training decreased the protein levels of ETC complex II, while those of other mitochondrial proteins, such as citrate synthase and cytochrome *c* were not altered in the skeletal muscle. Similar findings were obtained with EPI-rich cocoa products, where the supplementation did not improve exercise performance [152,153,154]. It seems that protective effects of EPI might be more pronounced under stressed conditions, as shown in H_2_O_2_ injured C2C12 cells [149]. Considering its properties, which improve exercise capacity mediated by upregulated muscular vascularization and mitochondrial biogenesis, EPI might be useful as a complement for medical conditions causing muscle weakness [147]. Upregulated capillarity by EPI administration (1 mg/kg, twice a day for 30 days) was accompanied by increased VEGF-A and decreased TSP1 levels in skeletal muscle of rats with congenital low running capacity. Along with the enhanced angiogenesis, myogenesis was also improved by EPI. The P38 MAPK signaling pathway, an important player of skeletal muscle differentiation, and MEF2A, a transcription factor for regulating muscle gene expression, were upregulated by EPI treatment. Increased mitochondrial biogenesis, mitochondrial volume, cristae abundancy, and COX were found together with increased expression levels of PGC1α, PGC1β, and TFAM. Interestingly, these beneficial effects lasted 15 days after EPI was discontinued.

Mitochondrial dysfunction has been proposed as a causative factor for the dysfunction of β cells and insulin resistance in type 2 diabetes [155]. Other evidence for the regulation of mitochondrial function by EPI was verified by the study with INS-1 832/13 rat β cells [149]. Glucose-stimulated insulin secretion from β cells was accompanied by upregulated protein levels of OXPHOS complexes including COX, and the consequently increased mitochondrial respiration following 10 μM EPI treatment for 24 h. The increased amounts of OXPHOS complexes by EPI were observed in adipocytes as well. The levels of complex II subunit A, complex V (subunit not specified in the paper), and COX subunit I were remarkably increased by 100 nM EPI treatment for 72 h. Enhanced mitochondrial biogenesis by EPI was confirmed by increased mitochondrial volume and its related regulators, such as SIRT1/3, PGC1α, NRF1/2, and TFAM [150]. Furthermore, EPI was able to reverse aging related changes in endothelial cells. Increased activity and amount of senescent biomarker β-galactosidase and decreased vasodilator nitric oxide and SIRT1 in aged cow endothelial cells were reversed by EPI treatment [129]. Declined mitochondrial biogenesis, along with downregulated ETC complex II, complex V, and COX along with TFAM and mitofilin in aged cells, were recovered to close to the levels in young control cells following incubation with 1 μM EPI for 48 h. Not only molecular profiles, but also physiological function of the endothelium was improved by EPI, since systolic blood pressure decreased due to increased vasodilation in aged rat aorta after EPI administration (1 mg/kg per day for 15 days).

The beneficial effects of EPI on mitochondrial function have also been used to sensitize cancer cells to radiotherapy using cultured cells [151]. After a 20 μM EPI treatment for 1 h, human pancreatic cancer cell lines (Panc-1, MIA PaCa-2) and a glioblastoma cell line (U87) were more sensitive to radiation and cell death. These changes were not observed in a normal fibroblast cell line (HNF). The cytotoxic effect on cancer cells was further confirmed by increased p21 production and Chk2 phosphorylation in Panc-1, but not in HNF; therefore, the sensitization by EPI was selective for cancer cells. This sensitization may be the result of enhanced mitochondrial function, since COX activity in Panc-1 but not in HNF cells was increased by EPI in a dose-dependent manner.

Notably, EPI modulates COX activity not only by increasing its biogenesis, but also by a more rapid process, likely through cell signaling and post-translational modifications, which has not been yet studied. For example, COX activity of healthy rat plantaris muscle was increased by incubation with 20 μM EPI for 25 min, which is insufficient time for the biosynthesis of COX, therefore another regulatory mechanism for COX and triggered by EPI must exist [147]. Interestingly, COX activity in rats with congenitally low running capacity did not respond to the short EPI treatment, which might be due to a faulty signaling pathway system in these animals. Increased EPI-mediated COX activity by mechanisms other than biogenesis was reproducibly shown in healthy mouse quadriceps under the same EPI treatment conditions [148]. A short-term effect on COX was also observed with a different type of catechin, epigallocatechin-3-gallate (EGCG) [156]. EGCG treatment increased COX activity, the mitochondrial membrane potential, mitochondrial respiration, and ATP content in primary human astrocyte and neuronal cell cultures. The effect of EGCG was not mediated by increased mitochondrial biogenesis since no change in the mRNA levels of mitofusin 2, TFAM, and PGC1α was observed. Further studies are needed to confirm the mechanism of short-term regulation by catechins on COX.

## 4. Betaine

Betaine (glycine betaine, *N*,*N*,*N*-trimethylglycine, BET, Figure 1C), a trimethyl derivative of the amino acid glycine, is a natural compound that is found ubiquitously in animals, plants, and microorganisms [157]. It is known that accumulation of BET improves the growth and survival of plants by protecting cellular components from abiotic stress such as drought, high salinity, and cold. BET is synthesized by betaine aldehyde dehydrogenase from betaine aldehyde, which is a derivative of choline [158]. It was first discovered in the nineteenth century as a byproduct of sucrose production from sugar beets (*Beta vulgaris*) [159]. The pool of BET in the body is mediated by synthesis through choline oxidation in hepatic and renal mitochondria as well as dietary intake [160], and is present mainly in the kidneys, liver, and brain [161]. Several studies have shown that the dietary intake of BET ranges from 100 to 400 mg/day, and the resting plasma BET concentration reaches 0.02–0.07 mmol/L in humans [162,163]. This compound is chemically stable and nontoxic, even at high concentrations, which broadens the potential for therapeutic uses.

The zwitterionic quaternary ammonium [(CH_3_)_3_N^+^ CH_2_COO^–^] form allows BET to exert several physiological functions. The zwitterionic characteristics and high solubility in water allow BET to function as an osmolyte. Concentrations can go as high as the millimolar ranges with no detrimental effect on the cells [159]. Additionally, it functions as a methyl group donor in several metabolic pathways. For example, in the methionine cycle BET donates a methyl group to homocysteine [160]. During such processes, BET converts into *N*,*N*-dimethylglycine and is then transformed into the amino acid glycine through a series of biochemical steps. Unlike polyphenols such as RSV and EPI, which can interact and neutralize free radicals directly and also modulate anti-/pro-oxidative enzymes, the anti-oxidative property of BET results from metabolites of the methionine cycle, such as methionine and S-adenosylmethionine (SAM), which play roles as anti-oxidants [164]. Additionally, it functions as a chaperone to protect proteins and DNA from denaturation under high concentrations of urea and NaCl in the medulla of the kidney [159]. In cells, BET does not bind to but is excluded from the hydration shell of a protein. This causes a thermodynamic force to fold the protein more compactly in order to decrease the amount of excluded water, due to the so-called osmophobic effect; therefore, BET is able to stabilize the protein’s native form.

The remarkable ability of BET to protect cells from exogenous stress factors, such as drought, high salinity, and high temperature has been extensively studied from microbial cells to mammals. Because of its low cost, safety, and effectiveness over a wide range of doses, BET has been frequently applied in animal husbandry on pigs, poultry, and lambs [165]. Supplementation of BET has positive effects on growth and feed efficiency in pigs and on egg production and body gain in chickens and turkeys [166,167,168,169,170], and these beneficial effects have been further demonstrated under heat stress conditions [171,172]. In addition, it has been fed to young salmon in fish farms to maintain osmotic balance and stimulate growth when they migrate to the sea [173]. As with animals, BET is beneficial for plant life. It improves growth, photosynthesis, nutrient uptake, and protects cells from chemical toxicity by reducing oxidative stress damage and excessive heavy metal uptake [174]. A positive role for BET in cold acclimation in plants has also been reported [175,176]. Not only multicellular organisms, but also unicellular bacteria benefit from BET. Microbial strains used for fermentation of lactate, ethanol, lysine, pyruvate, and vitamin B_12_ have been reported to derive benefit from BET, which not only provides a methyl group during biosynthetic processes, but also protects the cells against abiotic stress [177,178,179,180]. Unlike the studies with livestock, the benefits of BET in human subjects seem to be limited (see review on clinical trials in [181]). BET supplementation did not alter either BMI or body weight, but reduced total body fat mass and body fat percentage.

BET has been applied for therapeutic use in humans. Currently, it is used to treat homocystinuria, a systemic disorder with vascular complications and neurodegeneration [182,183] that is caused by a rare genetic disorder of methionine metabolism or deficiency of vitamin B_6_, B_12_, or folate [184]. Another possible application for BET was suggested for the treatment of nonalcoholic fatty liver diseases (hepatic steatosis, nonalcoholic hepatitis, and liver fibrosis) and alcoholic liver disease based on studies with rodents [185,186]. In animal models, these conditions share several histological and functional disturbances such as triglyceride accumulation, hyper homocysteinemia, dysregulation of lipid metabolism, insulin resistance, ER stress, mitochondrial dysfunction, and others [187,188] and often co-exist in clinical practice [189]. Multiple studies indicate that BET administration not only arrests but also in some cases reverses the progression to liver dysfunction [159,190,191,192]. Indeed, fruits of *Lycium barbarum*, a shrub native to China, which contain high amounts of BET has been traditionally used for the treatment of liver diseases in Asian countries [193]. However, the benefit of BET on nonalcoholic steatohepatitis in humans is inconclusive due to limitations with the clinical trial study design (see reviews on clinical trials in [194,195]). Other studies evaluating the effect of BET on cancer, diabetes, obesity, and Alzheimer’s disease revealed that BET consumption could lower the risk of those diseases [157,196].

Other useful applications of BET are its use as an additive in polymerase chain reaction (PCR) [197], cryopreservation [198], and personal care products such as toothpaste and moisturizers [182].

BET has been suggested to have effects on mitochondrial function. Endurance performance and resistance-based exercises such as running, squat repetitions, bench press throw, and vertical jumps, which are metabolically demanding tasks, were improved after BET administration [199,200,201,202]. Although the exact underlying mechanism was not investigated in those studies, one can speculate that the improved performance may be the result of enhanced mitochondrial energy metabolism.

Not much is known about a molecular target of BET for mitogenesis. Although PGC1α was upregulated by BET in db/db mice and rats with alcohol treatment, its upstream regulator and effect on mitogenesis were not evaluated in the studies [186,203,204]. Recently it was suggested that enhanced mitogenesis by BET is mediated by the SIRT1-PGC1α pathway [205]. Increased mitochondrial protein amount and ATP content by BET treatment were accompanied by increased mRNA and protein levels of SIRT1, PGC1α, NRF1, TFAM, and phosphorylated AMPK in C2C12 cells.

Several studies indicate that BET increases activity of mitochondrial ETC complexes, especially under heat stress. As discussed above, BET feeding was proven to be useful in animal husbandry, especially at high ambient temperatures, which normally have a deleterious impact on the activities of ETC complex I, III, and COX [206,207,208]. Chickens housed at over 32 °C presented with inhibited activities of complexes I + III and COX in liver [206] and those of complex I, III, and COX in skeletal muscle [207,208]. BET supplementation rescued the declined complex I activity caused by thermal stress [208]. In this study, BET also upregulated complex I and III activity in the non-thermal stress control group; however, complex II and COX were not affected. Hepatoprotection from chronic alcoholic damage by BET administration (1% *w*/*v* added to the diet for 4–5 weeks) in rats was accompanied with increased amounts of OXPHOS complexes [209]. Significantly increased steatosis and loss of intact complexes I, III, V, and COX, but not complex II, caused by ethanol treatment was rescued by BET. However, BET supplementation in control animals showed no effect (Table 3). Increased amounts of COX holoenzyme and of COX subunit I by BET were confirmed by one- and two-dimensional blue native gels and Western blot analysis, respectively. These results suggest that the protective effect of BET on liver from deleterious alcohol exposure may operate through maintaining the ability of mitochondrial OXPHOS.

BET has been proposed to protect cells from other stressed conditions as well. Ganesan et al. showed heart ETC complexes could be beneficiaries of BET treatment when the cells were under stress [210]. Isoprenaline-induced myocardial infarction in rats presented with decreased energy levels and activities of complexes I, II, and COX. Interestingly, pre-administration of BET (250 mg/kg per day for 30 days) was able to prevent the deleterious action of isoprenaline and rescued the ATP levels and the activities of complexes I, II, and COX to near control levels. BET treatment in control animals also showed a tendency towards increased ATP levels and ETC complexes activities, although they were not statistically significant. Reduced OXPHOS gene expression, mitochondrial DNA copy number, and TFAM amount in chicken liver with corticosterone treatment were improved by in ovo injection of BET [211]. Downregulated mRNA levels of COX I, II, III along with subunits of complexes I, III, V, which are encoded by the mitochondrial genome, were improved by BET, as well as COX activity. However, the expression level of the COX IV gene, which is encoded by the nuclear genome was not affected by either corticosterone or EPI.

More evidence for COX as a target of BET was suggested in mouse hepatocyte cell line H2.35 [212]. COX activity was increased by BET treatment for 30 min in a dose-dependent manner up to 2 mM, which resulted in elevated mitochondrial respiration, mitochondrial membrane potential, and cellular energy ATP. However, BET is not an allosteric regulator of COX, since BET treatment of purified COX did not change the enzyme activity. The beneficial effects of BET on mitochondrial function and energy metabolism were proposed to be through a mechanism similar to that mediating its cytoprotective properties. Based on the finding that the AKT signaling pathway was activated by BET in human muscle [213] and that activated AKT in mitochondria induced an increase in COX activity in hypoxic cardiomyocytes [214], one could speculate that the AKT signaling pathway targets COX and increases COX activity, possibly through alterations in post-translational modifications.

## 5. Conclusions

This review summarized various studies validating the regulatory effects of the natural compounds derived from plants, RSV, EPI, and BET on COX. In conclusion, these three small molecules promote COX biosynthesis and activity.

Although not all compounds share commonalities in their structures—RSV and EPI belong to the polyphenols, while BET is a methyl derivative of glycine—they have similar bioactivity, such as anti-oxidative, anti-inflammatory, and anti-cancer properties. Many studies with cultured cells and lab animals have suggested that the administration of these compounds improves the physiological features and function of cells, organs, and organisms. However, more pronounced effects were obtained under challenged or stressed conditions. In many experimental systems, they were capable of mitigating, preventing, or even rescuing pathological conditions including cardiovascular disease, neurodegenerative disease, metabolic disease, cancer, and aging. They could ameliorate the impaired conditions by alleviating oxidative stress due to direct and indirect antioxidant effects and in addition, by enhancing mitochondrial function and biogenesis, which are often disrupted in pathological conditions.

**Figure 2:**
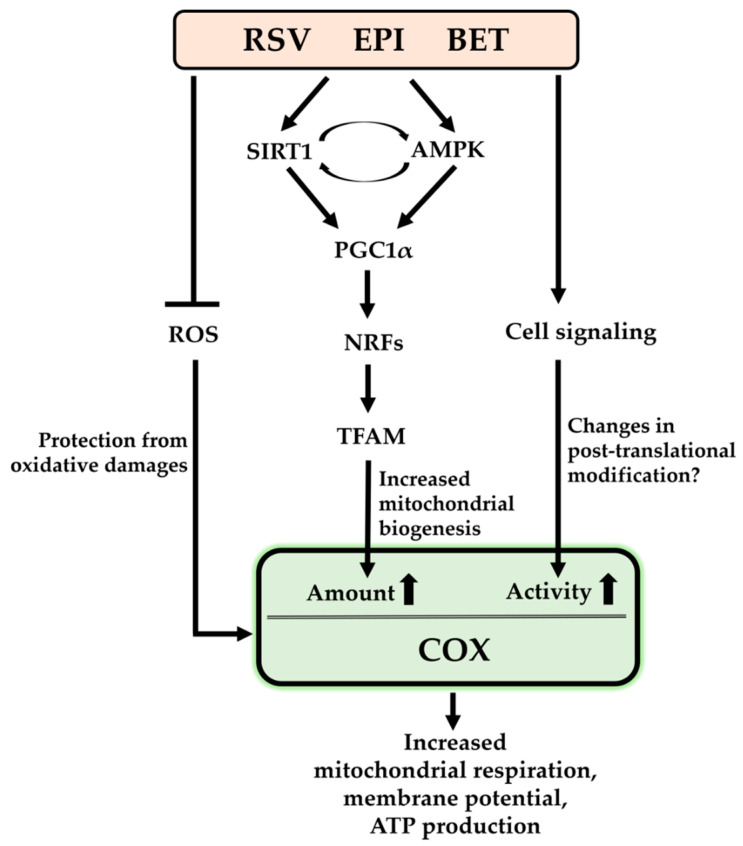
Regulatory mechanisms of resveratrol (RSV), (–)-epicatechin (EPI), and betaine (BET) on cytochrome *c* oxidase (COX): ROS, reactive oxygen species; SIRT1, sirtuin 1; AMPK, AMP activated protein kinase; PGC1α, peroxisome proliferator activated receptor gamma coactivator 1 alpha; NRFs, nuclear respiratory factors; TFAM, mitochondrial transcription factor A.

It has been shown that the increased mitochondrial biogenesis was accompanied by upregulation of biogenesis-related regulators, such as SIRT1, APMK, PGC1α, NRF1/2, and TFAM. In most cases, they increased the amount of COX and/or its enzymatic activity that result in improved mitochondrial function, as seen in increased mitochondrial respiration, mitochondrial membrane potential, and ATP levels (Figure 2). Although the studies did not investigate how crucial the changes in COX were for ameliorating pathological conditions, it is assumed that the improved mitochondrial function via enhanced COX levels and activity by the compounds might contribute to improving those conditions.

Interestingly, their stimulating effect on COX was not limited to protection from oxidative stress and the biogenesis. At least EPI and BET evidently increased COX activity in response to short term treatments. This enhancement was not facilitated by the increased protein levels but possibly by changes in post-translational modification, such as (de)phosphorylation of COX. In fact, several phosphorylation sites of COX have been identified and some of their effects on COX enzymatic activity have been verified. Although it is found that COX is regulated by the compounds, little is known about their regulatory mechanism on COX besides biogenesis and possibly post-translational modifications, and therefore further studies are necessary to investigate whether other regulatory mechanisms may exist. 

## Figures and Tables

**Table 1:** Effect of resveratrol (RSV) administration on cytochrome *c* oxidase (COX).

Reference	Experimental Subject	Experimental Condition	Result
[57]	Gastrocnemius muscle from 8-week-old male C57BL/6J mice with high fat diet	Supplementation of 400 mg/kg/day RSV to high fat diet for 15 weeks	Increased mRNA levels of COX Va, COX IV-1, COX Vb, COX VIIaL
[62]	Cultured C2C12 mouse myoblast cells	Incubation with 25 μM RSV for 24 h	Increased mRNA level of COX Vb
[62]	Gastrocnemius muscle and hepatocytes from C57BL/6J mice with high fat diet; WT mouse for SIRT1-KO model	Supplementation of 25~30 mg/kg/day RSV with high fat diet for 8 months	Increased mRNA level of COX Vb
[67]	Hippocampus from male Sprague Dawley rats with status epilepticus induced by kainic acid	Microinjection of 100 μmol RSV into hippocampus prior to kainic acid treatment	Increased COX I amount
[68]	Hippocampus from aged female Sprague Dawley rats induced by bilateral ovariectomy combined with intraperitoneal injection of D-galactose	Intragastric administration of 80 mg/kg/day RSV alone and in combination with isoflavones for 12 weeks	Increased COX I amount by RSV alone and in combination with isoflavones
[69]	Hippocampus, cerebral cortex, and cerebellum from 60-day-old male Wistar rats	Incubation with 0.1, 0.5, 5 mM RSV for 1 h in combination with citrulline or ammonia in brain homogenates	Increased COX activity in a dose-dependent manner; increased COX activity in the presence of citrulline by 0.1, 0.5 mM RSV in cerebral cortex and 5 mM RSV in hippocampus & increased COX activity in the presence of ammonia by 0.1, 0.5 mM RSV in cerebral cortex and cerebellum
[70]	Cultured human lymphoblastic leukemia cells overexpressed with Bcl-2; CEM/Bcl-2	Incubation with 10, 30, 50 μM RSV for 6 h	Decreased COX activity in a dose-dependent manner, while no change in COX I amount (50 μM RSV for 18 h)
[41]	Cultured human colon cancer cells; SW620	Incubation with 10 μM RSV for 48 h	Increased COX activity (~45%) and COX I amount

**Table 2:** Effect of (–)-epicatechin (EPI) administration on cytochrome *c* oxidase (COX).

Reference	Experimental Subject	Experimental Condition	Result
[144]	Cultured C2C12 mouse myoblast cells	Incubation with 10 μM EPI for 48 h	Increased COX I amount
[145]	Quadriceps femoris from detrained 5-month-old C57BL/6 male mice	Intragastric administration of 1 mg/kg of EPI twice a day during 14 days of detraining after 5 weeks of training	Increased COX activity (~108%) and COX II amount
[146]	Gastrocnemius muscle from hindlimb suspended 6-month-old male C57BL/6N mice	Intragastric administration of 1 mg/kg of EPI twice a day during 14 days of hindlimb suspension	Increased COX I amount
[147]	Plantaris muscle from 5-month-old male rats with congenital low running capacity	Intragastric administration of 1 mg/kg of EPI twice a day for 30 days	Increased COX II amount
[149]	Cultured rat β cells; INS-1 derived 832/13 cells	Incubation with 10 μM EPI for 24 h	Increased COX I amount
[150]	Cultured adipocytes excised from human subcutaneous adipose tissue	Incubation with 100 nM EPI for 72 h	Increased COX I amount
[129]	Cultured cow coronary artery endothelial cells with low passage number (young) and high passage number (aged)	Incubation with 1 μM EPI for 48 h	Increased COX I amount in both young and aged cells
[151]	Cultured Panc-1 pancreatic cancer cells	Incubation with 20, 50, 100, 200 μM EPI for 1 h	Increased COX activity in a dose-dependent manner (~59% by 200 μM EPI)
[147]	Plantaris muscle from healthy Sprague Dawley rats	Incubation with 20 μM EPI for 25 min	Increased COX activity
[148]	Quadriceps femoris muscle from 14-month-old male C57BL/6N mice	Incubation with 20 μM EPI for 25 min	Increased COX activity

**Table 3:** Effect of betaine (BET) administration on cytochrome *c* oxidase (COX).

Reference	Experimental Subject	Experimental Condition	Result
[209]	Livers from 45–48-day-old male Wister rats	Supplementation of 1% (*w*/*v*) BET to ethanol diet for 4–5 weeks	Increased amount of both COX holoenzyme and subunit I by BET in alcoholic liver, while no changes in controls
[210]	Hearts from Wistar male rats with myocardial infarction induced by isoprenaline	Intragastric administration of 250 mg/kg/day BET for 30 days prior to isoprenaline injection	Increased COX activity by BET in myocardial infarction, while no changes in controls
[211]	Livers from 8-week-old chickens injected with corticosterone	In ovo injection of 2.5 mg BET	Increased mRNA levels of COX I, II, and III and COX activity in corticosterone treated liver, while no changes in controls
[212]	Cultured H.2.35 mouse hepatocytes	Incubation of 0.5, 1, 2, 5, 10 mM BET for 30 min	Increased COX activity in a dose-dependent manner up to 2mM BET, while no change in activity of purified COX by incubation with EPI

## Abbreviations

RSV	resveratrol
EPI	(–)-epicatechin
BET	betaine
COX	cytochrome *c* oxidase
ETC	electron transport chain
OXPHOS	oxidative phosphorylation
ROS	reactive oxygen species
